# Supervised Exercise Training Improves 6 min Walking Distance and Modifies Gait Pattern during Pain-Free Walking Condition in Patients with Symptomatic Lower Extremity Peripheral Artery Disease

**DOI:** 10.3390/s21237989

**Published:** 2021-11-30

**Authors:** Stefano Lanzi, Joël Boichat, Luca Calanca, Lucia Mazzolai, Davide Malatesta

**Affiliations:** 1 Heart and Vessel Department, Division of Angiology, Lausanne University Hospital, 1011 Lausanne, Switzerland; joel.boichat@unisante.ch (J.B.); luca.calanca@chuv.ch (L.C.); lucia.mazzolai@chuv.ch (L.M.); 2 Institute of Sport Sciences, University of Lausanne, 1015 Lausanne, Switzerland; Davide.Malatesta@unil.ch

**Keywords:** intermittent claudication, vascular rehabilitation, 6 min walking test, functional walking

## Abstract

This study aimed to investigate the effects of supervised exercise training (SET) on spatiotemporal gait and foot kinematics parameters in patients with symptomatic lower extremity peripheral artery disease (PAD) during a 6 min walk test. Symptomatic patients with chronic PAD (Fontaine stage II) following a 3 month SET program were included. Prior to and following SET, a 6 min walk test was performed to assess the 6 min walking distance (6MWD) of each patient. During this test, spatiotemporal gait and foot kinematics parameters were assessed during pain-free and painful walking conditions. Twenty-nine patients with PAD (65.4 ± 9.9 years.) were included. The 6MWD was significantly increased following SET (+10%; *p* ≤ 0.001). The walking speed (+8%) and stride frequency (+5%) were significantly increased after SET (*p* ≤ 0.026). The stride length was only significantly increased during the pain-free walking condition (+4%, *p* = 0.001), whereas no significant differences were observed during the condition of painful walking. Similarly, following SET, the relative duration of the loading response increased (+12%), the relative duration of the foot-flat phase decreased (−3%), and the toe-off pitch angle significantly increased (+3%) during the pain-free walking condition alone (*p* ≤ 0.05). A significant positive correlation was found between changes in the stride length (r = 0.497, *p* = 0.007) and stride frequency (r = 0.786, *p* ≤ 0.001) during pain-free walking condition and changes in the 6MWD. A significant negative correlation was found between changes in the foot-flat phase during pain-free walking condition and changes in the 6MWD (r = −0.567, *p* = 0.002). SET was found to modify the gait pattern of patients with symptomatic PAD, and many of these changes were found to occur during pain-free walking. The improvement in individuals’ functional 6 min walk test was related to changes in their gait pattern.

## 1. Introduction

Lower extremity peripheral artery disease (PAD) is a chronic atherosclerotic vascular morbidity that leads to the narrowing and/or occlusion of lower-limb arteries [1]. PAD affects more than 200 million people worldwide [2]. Intermittent claudication—a pain occurring in the lower limbs during exercise and resolving with rest—is one of the typical manifestations of PAD [1]. Intermittent claudication has a huge impact on patients’ daily life activities, leading to reduced quality of life for these individuals [3,4].

Beyond the well-known manifestations of reduced walking capacities, physical function, altered muscular characteristics, and impaired balance [5,6,7,8,9], gait abnormalities have also been documented in patients with PAD [10,11,12,13,14,15,16,17]. Previous investigations reported a reduction in walking speed and cadence, smaller step length, and greater stance phase duration in patients with PAD compared to age-matched non-PAD individuals [13,17,18]. These changes were also observed during pain-free walking conditions [13,15,18]. The attributes of slower gait speed and stride frequency and shorter stride length were recently associated with higher levels of circulating biomarkers of inflammation and endothelial cell oxidative stress [19].

Cardiovascular risk management, pharmacological treatment, and exercise therapy are the main pillars of the treatment of PAD [1]. Following supervised exercise training (SET), greater treadmill walking performance has been well documented, with an improvement of ~82 m and ~120 m in treadmill pain-free and maximal walking distance, respectively [20]. Although less investigated, the 6 min walk test (6MWT)—an overground submaximal functional walking test—is also an effective tool that had been used to assess walking performance following interventions in patients with PAD [21,22]. A meta-analysis showed a mean improvement of ~35 m in 6 min walking distance (6MWD) following SET in individuals with PAD [23].

The question of whether SET induces gait changes, and whether the latter are related to treadmill performance in symptomatic patients with PAD, remains controversial [24,25,26,27,28,29,30]. To the best of our knowledge, the recent study by Lanzi et al. [29] is the only study to have assessed this relationship. Their study [29] showed that SET reduced the duration of the push-off and extended the duration of the foot-flat during a constant-speed treadmill exercise. Interestingly, in treadmill tests, gait changes were found to be significantly related to the delayed onset claudication distance [29]. However, the question of whether gait changes following SET are also related to functional walking improvements assessed by the 6MWT remains to be investigated. The investigation of this issue would be clinically relevant, as the 6MWT is representative of the type of walking one commonly partakes in daily life [21]. Following SET, a greater distance covered during the 6MWT is observed alongside an obvious increase in average walking speed. As spatiotemporal gait parameters (such as stride frequency and length) are influenced by walking speed [31], it is expected that gait pattern changes should be observed following SET during the 6MWT.

Previous investigations showed that the durations of the inner-stance phases (e.g., foot-flat and push-off) were altered during an acute treadmill exercise performed at a constant speed in patients with symptomatic PAD [29,32]. These findings showed that, compared to the pain-free walking condition, the duration of foot-flat phase increased and the duration of the push-off phase decreased with the onset of claudication pain [29,32]. The extended duration of the foot-flat phase during exertion may ameliorate the balance between oxygen supply and demand in the active ischemic calf musculature [13,29]. On the other hand, the reduced duration of the push-off phase during the transition from pain-free to painful walking may be related to exercise-induce ischemia, which may lead to calf muscle strength deficit and affect forward propulsion [8]. Notably, even if the onset of the claudication distance was delayed following SET, similar evolutions were observed during a constant-speed treadmill test regarding temporal gait parameters [29]. This suggests that once claudication is established and it worsens to moderate-to-maximal levels, similar gait adaptations occur during an acute bout of exercise before and following SET [29]. However, the evolution of spatiotemporal gait parameters during acute exercise in the form of the 6 min walk test following SET remain to be investigated in these individuals.

The primary aim of this study was to determine the spatiotemporal gait and foot kinematics parameters during an acute bout of 6MWT (acute adaptations) and in response to 3 month SET (chronic adaptations) in patients with symptomatic PAD. It was hypothesized that (1) SET would improve the 6 min walking distance (chronic adaptations); (2) SET would increase walking speed, and as well stride frequency and length (chronic adaptations); and (3) during the transition from pain-free to painful walking, similar acute gait adaptations would be observed during the 6MWT before and after SET.

## 2. Methods

### 2.1. Participants

Symptomatic patients with chronic lower extremity PAD were recruited from the Division of Angiology of the University Hospital of Lausanne, Switzerland. As described elsewhere, all the participants were enrolled in the Angiofit study and took part in the SET program [29,33]. For the purpose of this study, we included data regarding all the patients’ spatiotemporal gait and foot kinematics parameters during the 6MWT before and following SET. This study was approved by the local ethics committee (study number: 2016-01135) and was conducted in accordance with the Declaration of Helsinki. Before participation, the patients provided written, voluntary, informed consent.

### 2.2. Experimental Design

Each participant underwent (i) a pre-SET vascular medicine examination; (ii) a pre-SET 6MWT with gait assessment; (iii) a 3-month SET program; (iv) a post-SET 6MWT with gait assessment; and (v) a post-SET vascular medicine examination.

#### 2.2.1. Vascular Medicine Examination

The medical history of each individual was assessed, and physical and vascular evaluations were performed. The ankle–brachial index (ABI) and toe–brachial index (TBI) were measured in accordance with the guidelines [1]. ABI and TBI values related to the most symptomatic leg were considered for the analyses.

#### 2.2.2. Six Min Walk Test

In an indoor 50 m corridor, the patients were asked to walk as far as possible within 6 min to determine their 6MWD [34]. The patients were told that they were allowed to stop during the test and/or lean against the wall. If they did so, they were instructed to resume walking as soon as they could. During the test, standard phrases of encouragement were used in accordance with the guidelines [34]. The pain-free walking time (PFWT_6min_) and distance (PFWD_6min_) during the 6MWT was recorded during the test. These values correspond to the time or distance covered by the patients until the onset of pain. At the end of the test, the rate of perceived exertion on Borg’s scale (6: “very very light”; 20: “maximal effort”) [35] and the claudication pain severity on the visual analogue scale (VAS; 0: “no pain”; 10: “maximal pain”) were also recorded. In the post-SET condition, the 6MWT was performed at least 48 h following the last training session.

#### 2.2.3. Multimodal SET

The patients participated in the clinical multimodal SET program, as previously described [29,33,36,37]. Briefly, the patients performed Nordic walking twice weekly and exercises to strengthen the lower limbs once a week. Each exercise session’s duration was 60 min. However, this was the total time available for each training session and does not represent the actual exercising time performed by the patients. Indeed, depending on the exercise tolerance and the baseline functional status of the patient, the actual exercising time at the beginning of the program was around 15–25 min, which increased progressively up to 30–45 min at the end of the program. Each training session started with a 5–10 min warm-up and ended with a 5 min cool down. A clinical exercise physiologist supervised all of the training sessions.

During the outdoor Nordic walking sessions, the patients were asked to walk until they experienced moderate-to-severe claudication leg pain. Subsequently, the patients were asked to rest until they experienced complete (or almost complete) resolution of the pain. To enable complete supervision over the training sessions, patients were asked to walk back and forth over a 100–200 m section of level ground. In addition, the training intensity of the exercise sessions was also monitored using Borg’s scale [35]. During the first few weeks of training, patients were asked to exercise at a low intensity (9–11 on Borg’s scale). Subsequently, if feasible and safe, the exercise intensity was increased to a moderate or moderate-to-vigorous intensity (12–16 on Borg’s scale). The duration of each walking bout depended on the exercise intensity and the induced claudication pain. In general, walking bouts 5–10 min in duration were performed when the exercise intensity (assessed by Borg) was set at a low-to-moderate intensity. On the other hand, walking bouts 1 to 4 min in duration were performed when the exercise intensity was set at a moderate-to-vigorous intensity. The latter, despite inducing a higher cardiovascular stimulation, usually elicits a rapid increase in, and high levels of, claudication pain in these individuals.

The strengthening of the lower limbs was performed indoors with circuit training composed of 5–6 stations. Each station consisted of (1) a different type of walking, such as toe/heel, high knees, side-to-side, or backward walking, or (2) lower-limb resistance exercises (calf/heel raise, lunges, or squats) using body weight, dumbbells, or elastic bands. During the first few weeks of training, the patients were asked to perform 5–15 repetitions of each exercise using their body weight, interspersed with 30 to 60 s of recovery. The exercise training intensity was mainly set at a low level. In the following weeks, the patients were encouraged to exercise at a moderate intensity (12–14 on Borg’s scale). To that end, the patients were asked to increase the number of repetitions (20–30 repetitions using their body weight) or to exercise using dumbbells or elastic bands (10–20 repetitions).

During the program, the patients received 6 h of therapeutic workshops on cardiovascular risk factors and a healthy lifestyle (regarding nutrition, physical activity, and tobacco). Compliance with the SET program was defined by the percentage of attended sessions out of the total number of sessions [29].

### 2.3. Spatiotemporal Gait and Foot Kinematics Parameters

During the 6MWT, the patients wore two Physilog^®^ (GaitUp, Lausanne, Switzerland) inertial sensor units (dimensions: 50 mm × 40 mm × 16 mm, weight: 36 g) [38,39]. These sensors were used to evaluate spatiotemporal gait and foot kinematics parameters [38,39]. Physilog^®^ units integrate a microcontroller, a memory unit, a three-axial accelerometer (range ±3 g), a 3-axial gyroscope (range ±800° s^−1^), and a battery [38,39]. The inertial sensors displayed good accuracy and precision parameters and showed excellent test–retest reliability [39]. Physilog^®^ units have been validated in young [39] and older adults [38,39]. In addition, these sensors were also validated in individuals affected by stroke [40] and in children with cerebral palsy [41]. Finally, these sensors were previously used in other clinical populations, such as patients with Parkinson’s disease [42] and in patients with PAD [29].

The spatiotemporal gait and foot kinematics parameters were recorded during the whole 6MWT. For the analyses, ten consecutive strides were selected 1 min after the beginning of the test (pain-free walking: pain-free) and before the end of the 6 min walk test during the painful walking condition (pain).

During the 6MWT, walking speed, spatiotemporal gait, and foot kinematics parameters were assessed. Stride length was the only spatial parameter. The temporal parameters were stride duration and frequency (i.e., cadence) and the relative duration of the swing, stance, and double support phases (% of gait cycle duration). In addition, the relative duration of the inner-stance phases (i.e., loading response, foot-flat and push-off) were also reported [38,39]. The foot kinematics parameters were the heel-strike pitch angle (the positive angle formed between the level ground and the foot during heel-strike), the toe-off pitch angle (the negative angle formed between the level ground and the foot during toe-off), and the foot clearance (the foot’s height during the swing phase). Details regarding the estimation of the spatiotemporal gait and foot kinematics parameters are presented in the supplementary materials.

The symmetry between the legs was assessed by dividing the values of the most symptomatic leg by those of the less or non-symptomatic leg [29].

### 2.4. Statistical Analysis

The sample size was estimated using our previous data [36], showing that 23 patients were necessary (power 80%; α = 5%). The Kolmogorov–Smirnov test was used to assess the normality of the distribution. First, a two-way repeated measures analysis of variance (ANOVA) (time (before SET vs. after SET) × duration (pain-free vs. pain)) was used to evaluate the symmetry of the spatiotemporal gait and foot kinematics parameters between legs. Second, a two-way ANOVA was also used to compare the gait pattern in the most symptomatic leg alone. If the ANOVAs showed a significant main effect (time or duration) or interaction effect (time × duration), multiple comparison analyses with Bonferroni adjustments were performed to detect the differences. Paired t-tests were used to compare the 6MWT and vascular parameters before and following the multimodal SET program. To determine the relationship between the spatiotemporal gait and foot kinematics changes (i.e., delta; post- minus pre-training values) and changes in 6MWD following SET, partial correlations, controlled for gait baseline values, were performed. The data are expressed as the mean ± SD. The level of significance was set at *p* ≤ 0.05. SPSS 27 software (IBM Corporation, Armonk, NY, USA) was used for the statistical analyses.

## 3. Results

### 3.1. Participants

Twenty-nine symptomatic patients with chronic PAD were included. All the patients completed the 3 month SET program. Their general characteristics are reported in Table 1. A similar pharmacological therapy was observed before and after SET, except that one patient started antidiabetic therapy during SET. The compliance of the participants with the SET program was 98 ± 4%.

### 3.2. Vascular Parameters

The values regarding the ABI (before SET: 0.79 ± 0.14 after SET: 0.78 ± 0.14; *p* = 0.829) and TBI (before SET: 0.60 ± 0.15, after SET: 0.60 ± 0.18; *p* = 0.971) were unchanged following SET.

### 3.3. Six Min Walk Test

Following SET, a significant increase was observed in the 6MWD values (+10%; Table 2). The values regarding PFWT_6min_ and PFWD_6min_ did not change significantly (Table 2). The RPE at the end of the 6MWT was significantly higher after SET (Table 2). Values relating to claudication pain at the end of the 6MWT were unchanged (Table 2).

### 3.4. Spatiotemporal Gait and Foot Kinematics Parameters: Acute and Chronic Adaptations

The symmetry of the spatiotemporal gait and foot kinematics parameters between legs showed no significant time, duration, or time × duration interaction effect (data not shown). This suggests that similar acute and chronic adaptations were present in both legs of the participants. Therefore, for sake of clarity, only the results regarding the most symptomatic leg were presented.

#### 3.4.1. Spatiotemporal Gait Parameters (Acute Adaptations)

During the 6MWT, a significant duration effect was observed for all the spatiotemporal gait parameters (Table 3). Multiple comparison analyses demonstrated that walking speed, stride duration, stride frequency, stride length, duration of swing, loading response duration, and push-off phase duration significantly decreased during the transition from the pain-free to painful walking condition, whereas the duration of the stance, foot-flat, and double support phases significantly increased during the 6MWT (Table 3).

#### 3.4.2. Spatiotemporal Gait Parameters (Chronic Adaptations)

##### Walking Speed

A significant time and time × duration interaction effect was found with regard to the walking speed (Table 3). Multiple comparison analyses revealed that walking speed was significantly increased following SET during the pain-free and painful walking conditions (*p* ≤ 0.026, Table 3).

##### Stride Length

A significant time and time × duration interaction effect was found regarding the stride length (Table 3). Multiple comparison analyses showed that stride length was significantly increased following SET (time effect: *p* = 0.013). Compared to the values recorded before SET, stride length was significantly increased following SET during the pain-free walking condition alone (*p* = 0.001), whereas no significant differences were observed during the painful walking condition (*p* = 0.569).

##### Stride Duration and Frequency

Following SET, stride duration and frequency significantly increased (time effect: *p ≤* 0.001) with no significant time × duration interaction effect (Table 3).

##### Stance and Swing Phase

After SET, the relative duration of the stance and swing phase was unchanged (time effect: *p* = 0.431); however, a significant time × duration interaction effect was observed (Table 3). Multiple comparison analyses revealed a significant increase in the relative duration of the stance phase during the painful condition compared to the pain-free condition following SET alone (*p* = 0.008). Similarly, a significant decrease in the relative duration of the swing phase was observed during the painful condition compared to the pain-free walking condition following SET alone (*p* = 0.008).

##### Inner-Stance Phases

A significant time and time × duration interaction effect was observed regarding the relative duration of the loading response (Table 3). Multiple comparison analyses revealed a significant increase in this parameter following SET (time effect: *p* = 0.013). After SET, there was a significant increase in the relative duration of the loading response during the pain-free walking condition alone (*p* = 0.001), whereas no significant differences were observed during the painful walking condition (*p* = 0.523).

The relative duration of the foot-flat phase was unchanged after SET (time effect: *p* = 0.139); however, a significant time × duration interaction effect was observed (Table 3). Multiple comparison analyses revealed a significant decrease following SET during the pain-free walking condition alone (*p* = 0.002), whereas no significant differences were observed during the painful walking condition (*p* = 0.420).

No significant time and time × duration interaction effect was observed for the relative duration of the push-off or for the double support phases (Table 3).

### 3.5. Foot Kinematics Parameters (Acute Adaptations)

During the 6MWT, all the foot kinematics parameters showed a significant duration effect, except for the first maximal toe clearance (Table 4). Multiple comparison analyses showed that the heel-strike pitch angle, toe-off pitch angle, maximal heel clearance, second maximal toe clearance, and minimal toe clearance significantly decreased during the transition from the pain-free to the painful walking condition during the 6MWT (Table 4).

### 3.6. Foot Kinematics Parameters (Chronic Adaptations)

The toe-off pitch angle was unchanged after SET (time effect: *p* = 0.356); however, a significant time × duration interaction effect was observed (Table 4). Multiple comparison analyses revealed that, compared to before SET, the toe-off pitch angle significantly increased following SET during the pain-free walking condition alone (*p* = 0.05), whereas no significant differences were observed during the painful walking condition (*p* = 0.938). No significant time and time × duration interaction effect was observed regarding the heel-strike pitch angle, maximal heel clearance, second maximal toe clearance, or minimal toe clearance (Table 4).

## 4. Correlations

The relationships between gait pattern changes during the pain-free walking condition and changes in 6MWD following SET are displayed in Table 5. A significant positive correlation was found between changes in stride length, stride frequency, and second max toe clearance during the pain-free walking condition and changes in 6MWD (Table 5). On the other hand, a significant negative correlation was found between changes in the duration of the foot-flat phase during the pain-free walking condition and changes in 6MWD (Table 5).

## 5. Discussion

The results of this study partially confirm our hypotheses: (1) SET improved the 6MWD in patients with symptomatic PAD; (2) following SET, walking speed, stride frequency and stride length were significantly greater during the 6MWT. However, stride length was significantly increased following SET during the pain-free walking condition alone, whereas no significant differences were observed during the painful walking condition. Similarly, changes in the relative duration of the inner-stance phases (loading response and foot-flat) and the toe-off pitch angle following SET were observed during the pain-free walking condition alone; (3) during the transition from the pain-free to the painful walking condition, the spatiotemporal gait and foot kinematics parameters were shown to undergo a similar evolution before and after SET during the 6MWT. Finally, our results showed that changes in stride length and frequency and in the relative duration of the foot-flat phase during the pain-free walking condition were related to changes in functional walking performance during the 6MWT following SET.

The results of the present investigation confirm previous findings, which showed that SET improves 6MWD in symptomatic patients with PAD [9,23]. We observed a ~43 m improvement in 6MWD, which was greater than the substantial meaningful change of +20 m [43] or +35 m [44] previously observed in these individuals. The greater improvement in 6MWD observed in the present investigation may have been related to the training characteristics of the multimodal SET program. Indeed, the patients combined the strengthening of the lower limbs with Nordic walking, which are both functional training modalities. This type of training likely led to better improvements in functional walking performance. The greater improvement in 6MWD could also be related to the 50 m course length, as previous studies showed that longer course lengths were associated with greater walking distances [45]. By contrast, this improvement was similar to the minimal detectable change of >46 m recently observed in patients with claudication [46]. Taken together, these results suggest that multimodal SET is effective at improving functional walking performance in patients with symptomatic PAD [33,36,37].

During the transition from the pain-free to painful walking conditions, similar acute adaptations were observed for the spatiotemporal gait and foot kinematics parameters during the 6MWT before and after SET. These results extend previous findings observed during constant-speed treadmill exercises in patients with symptomatic PAD [13,29] and highlight that similar acute gait adaptions also occur during the 6MWT, which is a more functional form of walking that represents daily life more accurately [21]. Previous studies have shown that, when compared to aged-matched individuals, gait abnormalities exist from the first step taken (pain-free), suggesting muscle metabolic myopathy in patients with PAD [15,47,48]. Gait worsening was also documented once leg claudication pain was established, highlighting the role of muscle ischemia on gait pattern changes during exertion in these individuals [15]. Our results are in line with these findings. We observed that the walking speed, stride duration, stride frequency, stride length, relative duration of swing, loading response duration, and push-off phase duration decreased (pain-free > end), whereas the duration of the stance, foot-flat and double support phases significantly increased (pain-free < end) during the transition from the pain-free to the painful walking condition during the 6MWT. The extended duration of the stance and the foot-flat phases during exertion may ameliorate the balance between oxygen supply and demand in the active ischemic calf musculature [13,29]. It is also possible that patients adopt this pattern to improve their stability during painful walking [13,29]. The reduced duration of the push-off phase during the transition from the pain-free to the painful walking condition may be related to exercise-induced ischemia, which may lead to calf muscle strength deficit and affect forward propulsion [8]. Consequently, this may also affect walking speed, stride frequency and stride length, and foot kinematics, especially during the 6MWT, where patients are allowed to choose their own walking pace.

In current research, there are a limited number of studies regarding gait pattern changes following exercise interventions in patients with symptomatic PAD, and the findings are inconsistent. Indeed, some studies [27,29,30,49], but not others [24,25,28], observed significant gait changes following SET. The results of the present investigation showed that gait pattern was modified in patients with symptomatic PAD during the 6MWT following multimodal SET. It is, however, interesting to note that many of these changes occurred during the pain-free walking condition alone. Indeed, although walking speed and stride frequency increased following SET, stride length was significantly increased following SET during the pain-free walking condition alone, whereas no significant differences were observed during the painful walking condition. These findings indicate that the increased walking speed observed during the painful walking condition following SET is mainly related to an increased stride frequency rather than increased stride length. Similarly, the relative duration of the loading response phase increased, and the relative duration of the foot-flat phase decreased following SET during the pain-free walking condition alone. The toe-off pitch angle was also increased following SET, but again, during the pain-free walking condition alone. These observations are in contrast to previous findings that show the relative duration of the foot-flat phase was increased during constant-speed maximal treadmill exercises following SET in patients with symptomatic PAD [29]. A possible explanation is that this may have been due to the testing protocol used to assess gait changes following SET. Indeed, compared to the constant-speed treadmill test, walking speed during 6MWT exhibited different values before and after SET. Following SET, the patients demonstrated an improved dynamic balance, which allowed them to walk faster during the 6MWT, causing a reorganization of the durations of the loading response (increased) and foot-flat (decreased) phases during the pain-free walking condition. This is in line with previous observations, which showed that the duration of the foot-flat phase was negatively correlated to walking speed in both PAD and non-PAD individuals [13]. Once claudication pain began and worsened to moderate-to-maximal levels, the walking speed decreased during the 6MWT. Interestingly, the relative durations of the loading response and the foot-flat phases returned to the pre-SET values despite the greater walking speed in the post-SET condition. This suggests that factors other than the walking speed are related to gait pattern changes. These findings indicate the potential role of exercise-induced ischemia and claudication pain on gait adaptations during exertion in patients with symptomatic PAD.

The use of non-invasive inertial sensors with the aim to investigate gait pattern during physical assessment has potential applications with regard to the optimization of the prescription of training in patients with PAD. Indeed, these inertial sensors may easily assess gait pattern evolutions during functional acute exercise performed before and following an exercise training program. This technology allows one to evaluate the gait changes in the transition from the pain-free to painful walking condition, and therefore produces a valid description of daily-life walking pattern in these individuals. In addition, by evaluating the potential correlation between gait pattern and functional performance changes following rehabilitation, specific training approaches could be conceived to optimize patients’ benefits. Interestingly, our results showed that the changes in stride length and frequency during the pain-free walking condition were positively correlated to changes in 6MWD. In addition, the changes in the relative duration of the foot-flat phase during the pain-free walking condition were negatively correlated with changes in 6MWD. These findings suggest a link between changes in gait pattern during the pain-free walking condition and improved functional walking performance in patients with symptomatic PAD. These results feature important clinical implications and indicate the need for further investigations regarding the effects of specific gait training modalities on gait pattern and its relation to functional walking performance in these individuals. A previous meta-analysis showed that walking training with cueing of cadence improves spatiotemporal gait parameters more than walking training alone in older patients with cardiovascular disease [50]. Walking training with cueing of cadence, which was usually 5–10% greater than comfortable cadence, improves walking speed, stride length and frequency, and walking symmetry in patients who have experienced a stroke [50]. Based on the gait abnormalities previously observed in patients with PAD [13,15,17,18], these specific gait training modalities could be promising with regard to improving gait pattern and (functional) walking performance in these individuals.

This study featured some limitations. First, the present investigation lacked a control group that did not participate in the 3 month SET. Even though previous findings showed no difference in gait pattern over time in patients with PAD who did not participate in a vascular rehabilitation program [24], future randomized controlled trials are needed to better investigate gait changes following training interventions in these individuals. Second, because of the descriptive nature of our results, it was not possible to elucidate the mechanisms related to our observations. More detailed kinetics and kinematics gait analyses are needed to better describe gait pattern before and after SET. Third, even though it was used in previous works, the inertial system used in the present investigation has never been validated in patients with PAD.

In conclusion, these results show that multimodal SET modifies gait pattern during the 6 min walk test in patients with symptomatic PAD. However, many of these changes (stride length, the relative duration of the loading response and foot-flat phases, and toe-off pitch angle) only occurred during the pain-free walking condition, highlighting the role of claudication pain in gait pattern in this population. In addition, changes in stride length and frequency and in the relative duration of the foot-flat phase during the pain-free walking condition were correlated with changes in 6 min walking distance. These findings suggest that new rehabilitation strategies, including specific gait training modalities, should be further investigated in this population.

## Figures and Tables

**Table 1:** Characteristics of the participants.

Variables	Mean ± SD or *n* (%)
Number of included patients	29
Men	15 (52)
Women	14 (48)
Age—years	65.4 ± 9.9
BMI—kg·m^−2^	28.7 ± 6.2
Cardiovascular risk factors	
Hypercholesterolemia	23 (79)
Hypertension	24 (83)
Smoking (current)	12 (41)
Smoking (former)	13 (45)
Smoking (never)	4 (14)
Family history of CVD	13 (45)
Type 2 diabetes mellitus	8 (28)
Type 1 diabetes	1 (3)
Prior history of CVD	
Cardiac	8 (28)
Cerebrovascular	2 (7)
Prior arterial revascularisation	13 (45)
Ongoing treatment	
Antiplatelet	28 (97)
Antihypertensive	24 (83)
Lipid lowering	23 (79)
Antidiabetic	9 (31)

BMI: body mass index; CVD: cardiovascular disease.

**Table 2:** 6 min walk test before and after supervised exercise training.

Variable	Before	After	*p* Value
6MWD—m	425.5 ± 70.3	468.7 ± 84.3	**≤0.001**
PFWT_6min_—s	125.1 ± 55.4	123.3 ± 54.0	0.869
PFWD_6min_—m	162.5 ± 67.4	179.2 ± 77.6	0.245
6MWT_RPE_	12.3 ± 2.5	13.2 ± 2.2	**0.043**
6MWT_VAS_	6.8 ± 2.2	7.1 ± 1.8	0.432

6MWD: 6 min walking distance; PFWT_6min_: pain-free walking time during the 6 min walk test; PFWD_6min_: pain-free walking distance during the 6 min walk test; 6MWT_RPE_: rate of perceived exertion at the end of the 6 min walk test; 6MWT_VAS_: claudication pain at the end of the 6 min walk test. Bold *p* value is statistically significant (*p* ≤ 0.05).

**Table 3:** Spatiotemporal gait parameters in the most symptomatic leg during the 6 min walk test before and after supervised exercise training (SET).

	Before SET		After SET		Two-Way ANOVA *p*-Values		
Variable	Pain-Free	Pain	Pain-Free	Pain	Time Effect	Duration Effect	Time × Duration
Walking speed—m·s^−1^	1.3 ± 0.2	1.2 ± 0.2 *	1.5 ± 0.2 ^#^	1.3 ± 0.2 *^,^^#^	**≤0.001**	**≤0.001**	**0.031**
Spatial Parameter							
Stride length—m	1.4 ± 0.2	1.3 ± 0.2 *	1.4 ± 0.2 ^#^	1.3 ± 0.2 *	**0.013**	**≤0.001**	**0.020**
Temporal Parameters							
Stride duration—s	1.0 ± 0.1	1.1 ± 0.1	1.0 ± 0.1	1.0 ± 0.1	**≤0.001**	**≤0.001**	0.502
Stride frequency—Hz	1.0 ± 0.1	0.9 ± 0.1	1.0 ± 0.1	1.0 ± 0.1	**≤0.001**	**≤0.001**	0.297
Stance duration—%	60.1 ± 2.0	60.5 ± 1.9	59.6 ± 2.3	60.5 ± 2.4 ^£^	0.431	**0.017**	**0.033**
Swing duration—%	39.9 ± 2.0	39.5 ± 1.9	40.4 ± 2.3	39.5 ± 2.4 ^£^	0.431	**0.017**	**0.033**
Loading response—%	12.5 ± 3.1	11.0 ± 2.7 *	14.0 ± 3.2 ^#^	11.3 ± 3.0 *	**0.013**	**≤0.001**	**0.019**
Foot-flat—%	55.2 ± 6.3	59.8 ± 6.3 *	53.2 ± 6.4 ^#^	60.4 ± 6.3 *	0.139	**≤0.001**	**0.011**
Push-off—%	32.3 ± 4.9	29.2 ± 4.8	32.7 ± 5.5	28.3 ± 5.0	0.706	**≤0.001**	0.098
Double support—%	20.9 ± 3.5	22.9 ± 3.7	19.5 ± 4.0	21.9 ± 3.9	0.057	**≤0.001**	0.413

Ten consecutive strides were analyzed during pain-free walking (pain-free) and during painful walking at the end of the 6 min walk test (pain). Bold *p* value is statistically significant. * *p* ≤ 0.05 for significant difference compared to pain-free. ^#^ *p* ≤ 0.05 for significant difference compared to before SET. ^£^ *p* ≤ 0.05 for significant difference to pain-free within after SET condition.

**Table 4:** Foot kinematics in the most symptomatic leg during the 6 min walk test before and after supervised exercise training (SET).

	Before SET		After SET		Two-Way ANOVA *p*-Values		
Variable	Pain-Free	Pain	Pain-Free	Pain	Time Effect	Duration Effect	Time × Duration
Heel-strike pitch angle—°	26.9 ± 6.8	24.4 ± 6.2	27.8 ± 5.9	24.2 ± 5.0	0.463	**≤0.001**	0.080
Toe-off pitch angle—°	−68.8 ± 6.4	−65.2 ± 8.1 *	−70.5 ± 6.3 ^#^	−65.1 ± 7.2 *	0.356	**≤0.001**	**0.047**
Max heel clearance—cm	30.1 ± 5.4	28.9 ± 5.6	30.5 ± 4.5	29.2 ± 5.1	0.517	**0.003**	0.476
First max toe clearance—cm	7.8 ± 4.1	7.6 ± 4.2	7.9 ± 3.2	7.7 ± 3.5	0.837	0.252	0.895
Second max toe clearance—cm	18.1 ± 4.1	16.7 ± 3.4	17.5 ± 4.2	15.6 ± 3.6	0.082	**≤0.001**	0.210
Min toe clearance—cm	2.4 ± 0.9	2.1 ± 0.8	2.4 ± 1.1	2.2 ± 1.1	0.800	**0.013**	0.933

Ten consecutive strides were analyzed during pain-free walking (pain-free) and during painful walking at the end of the 6 min walk test (pain). Bold *p* value is statistically significant. * *p* ≤ 0.05 for significant difference compared to pain-free. ^#^ *p* ≤ 0.05 for significant difference compared to before SET.

**Table 5:** Relationship between spatiotemporal gait and foot kinematics changes during pain-free walking condition and changes in 6 min walking distance following supervised exercise training.

Gait Pattern Changes	Relationship with the 6 min Walking Distance Changes	*p* Value
Stride length—m	r = 0.497	**0.007**
Stride frequency—Hz	r = 0.786	**≤0.001**
Stance duration—%	r = −0.261	0.180
Swing duration—%	r = 0.261	0.180
Loading response—%	r = 0.320	0.097
Foot-flat—%	r = −0.567	**0.002**
Push-off—%	r = 0.303	0.116
Double support—%	r = −0.356	0.060
Heel-strike pitch angle—°	r = 0.313	0.105
Toe-off pitch angle—°	r = −0.100	0.614
Max heel clearance—cm	r = −0.112	0.570
First max toe clearance—cm	r = 0.035	0.858
Second max toe clearance—cm	r = 0.424	**0.025**
Min toe clearance—cm	r = −0.117	0.553

Bold *p* value is statistically significant (*p* ≤ 0.05). All correlations were controlled for gait baseline values.

## Data Availability

The data presented in this study are available on request from the corresponding author.

## Supplementary Materials

The following are available online at https://www.mdpi.com/article/10.3390/s21237989/s1.

## References

[1] Aboyans V., Ricco J.B., Bartelink M.E.L., Bjorck M., Brodmann M., Cohnert T. (2018). 2017 ESC Guidelines on the Diagnosis and Treatment of Peripheral Arterial Diseases, in collaboration with the European Society for Vascular Surgery (ESVS): Document covering atherosclerotic disease of extracranial carotid and vertebral, mesenteric, renal, upper and lower extremity arteriesEndorsed by: The European Stroke Organization (ESO) The Task Force for the Diagnosis and Treatment of Peripheral Arterial Diseases of the European Society of Cardiology (ESC) and of the European Society for Vascular Surgery (ESVS). Eur. Heart J..

[2] Song P., Rudan D., Zhu Y., Fowkes F.J.I., Rahimi K., Fowkes F.G.R., Rudan I. (2019). Global, regional, and national prevalence and risk factors for peripheral artery disease in 2015: An updated systematic review and analysis. Lancet Glob. Health.

[3] Liles D.R., Kallen M.A., Petersen L.A., Bush R.L. (2006). Quality of Life and Peripheral Arterial Disease. J. Surg. Res..

[4] Regensteiner J.G., Hiatt W.R., Coll J.R., Criqui M.H., Treat-Jacobson D., McDermott M.M. (2008). The impact of peripheral arterial disease on health-related quality of life in the Peripheral Arterial Disease Awareness, Risk, and Treatment: New Resources for Survival (PARTNERS). Program. Vasc. Med..

[5] Gardner A.W., Montgomery P.S. (2001). Impaired Balance and Higher Prevalence of Falls in Subjects with Intermittent Claudication. J. Gerontol. Ser. A Boil. Sci. Med. Sci..

[6] McDermott M.M., Liu K., Greenland P., Guralnik J.M., Criqui M.H., Chan C. (2004). Functional decline in peripheral arterial disease: Associations with the ankle brachial index and leg symptoms. JAMA.

[7] Mockford K.A., Mazari F.A., Jordan A.R., Vanicek N., Chetter I.C., Coughlin P.A. (2011). Computerized Dynamic Posturography in the Objective Assessment of Balance in Patients with Intermittent Claudication. Ann. Vasc. Surg..

[8] Schieber M.N., Hasenkamp R.M., Pipinos I.I., Johanning J.M., Stergiou N., DeSpiegelaere H.K. (2017). Muscle strength and control characteristics are altered by peripheral artery disease. J. Vasc. Surg..

[9] Treat-Jacobson D., McDermott M.M., Bronas U.G., Campia U., Collins T.C., Criqui M.H., Gardner A.W., Hiatt W.R., Regensteiner J.G., Rich K. (2019). Optimal Exercise Programs for Patients with Peripheral Artery Disease: A Scientific Statement From the American Heart Association. Circulation.

[10] Celis R., Pipinos I.I., Scott-Pandorf M.M., Myers S.A., Stergiou N., Johanning J.M. (2009). Peripheral arterial disease affects kinematics during walking. J. Vasc. Surg..

[11] Chen S.-J., Pipinos I., Johanning J., Radovic M., Huisinga J.M., Myers S.A., Stergiou N. (2008). Bilateral claudication results in alterations in the gait biomechanics at the hip and ankle joints. J. Biomech..

[12] Crowther R.G., Spinks W.L., Leicht A.S., Quigley F., Golledge J. (2007). Relationship between temporal-spatial gait parameters, gait kinematics, walking performance, exercise capacity, and physical activity level in peripheral arterial disease. J. Vasc. Surg..

[13] Gommans L.N., Smid A.T., Scheltinga M.R., Cancrinus E., Brooijmans F.A., Meijer K., Teijink J.A. (2017). Prolonged stance phase during walking in intermittent claudication. J. Vasc. Surg..

[14] Guilleron C., Beaune B., Durand S., Pouliquen C., Henni S., Abraham P. (2021). Gait alterations in patient with intermittent claudication: Effect of unilateral vs bilateral ischemia. Clin. Physiol. Funct. Imaging.

[15] Koutakis P., Johanning J.M., Haynatzki G.R., Myers S., Stergiou N., Longo G.M., Pipinos I.I. (2010). Abnormal joint powers before and after the onset of claudication symptoms. J. Vasc. Surg..

[16] Myers S.A., Huben N.B., Yentes J., McCamley J., Lyden E.R., Pipinos I.I., Johanning J.M. (2015). Spatiotemporal Changes Posttreatment in Peripheral Arterial Disease. Rehabilitation Res. Pract..

[17] Szymczak M., Krupa P., Oszkinis G., Majchrzycki M. (2018). Gait pattern in patients with peripheral artery disease. BMC Geriatr..

[18] Gardner A.W., Forrester L., Smith G.V. (2001). Altered gait profile in subjects with peripheral arterial disease. Vasc. Med..

[19] Gardner A.W., Montgomery P.S., Casanegra A.I., Silva-Palacios F., Ungvari Z., Csiszar A. (2016). Association between gait characteristics and endothelial oxidative stress and inflammation in patients with symptomatic peripheral artery disease. AGE.

[20] Lane R., Ellis B., Watson L., Leng G.C. (2014). Exercise for intermittent claudication. Cochrane Database Syst. Rev..

[21] McDermott M.M., Guralnik J.M., Criqui M.H., Liu K., Kibbe M.R., Ferrucci L. (2014). Six-Minute Walk Is a Better Outcome Measure Than Treadmill Walking Tests in Therapeutic Trials of Patients with Peripheral Artery Disease. Circulation.

[22] McDermott M.M., Guralnik J.M., Tian L., Zhao L., Polonsky T.S., Kibbe M.R., Criqui M.H., Zhang D., Conte M.S., Domanchuk K. (2020). Comparing 6-minute walk versus treadmill walking distance as outcomes in randomized trials of peripheral artery disease. J. Vasc. Surg..

[23] Parmenter B., Dieberg G., Smart N.A. (2015). Exercise Training for Management of Peripheral Arterial Disease: A Systematic Review and Meta-Analysis. Sports Med..

[24] Crowther R.G., Spinks W.L., Leicht A.S., Sangla K., Quigley F., Golledge J. (2008). Effects of a long-term exercise program on lower limb mobility, physiological responses, walking performance, and physical activity levels in patients with peripheral arterial disease. J. Vasc. Surg..

[25] Crowther R.G., Spinks W.L., Leicht A.S., Sangla K., Quigley F., Golledge J. (2009). The influence of a long term exercise program on lower limb movement variability and walking performance in patients with peripheral arterial disease. Hum. Mov. Sci..

[26] Dziubek W., Bulińska K., Stefańska M., Woźniewski M., Kropielnicka K., Jasiński T., Jasiński R., Pilch U., Dąbrowska G., Skórkowska-Telichowska K. (2015). Peripheral arterial disease decreases muscle torque and functional walking capacity in elderly. Maturitas.

[27] Haga M., Hoshina K., Koyama H., Miyata T., Ikegami Y., Murai A., Nakamura Y. (2020). Bicycle exercise training improves ambulation in patients with peripheral artery disease. J. Vasc. Surg..

[28] King S., Vanicek N., Mockford K.A., Coughlin P.A. (2012). The effect of a 3-month supervised exercise programme on gait parameters of patients with peripheral arterial disease and intermittent claudication. Clin. Biomech..

[29] Lanzi S., Boichat J., Calanca L., Aubertin P., Malatesta D., Mazzolai L. (2021). Gait changes after supervised exercise training in patients with symptomatic lower extremity peripheral artery disease. Vasc. Med..

[30] Schieber M.N., Pipinos I.I., Johanning J.M., Casale G.P., Williams M.A., DeSpiegelaere H.K. (2019). Supervised walking exercise therapy improves gait biomechanics in patients with peripheral artery disease. J. Vasc. Surg..

[31] Cavagna G.A., Willems P.A., Heglund N.C. (2000). The role of gravity in human walking: Pendular energy exchange, external work and optimal speed. J. Physiol..

[32] Gommans L.N., Fokkenrood H.J., van Dalen H.C., Scheltinga M.R., Teijink J.A., Peters R.J. (2015). Safety of supervised exercise therapy in patients with intermittent claudication. J. Vasc. Surg..

[33] Lanzi S., Calanca L., Berchtold A., Mazzolai L. (2021). Improvement in 6-Minute Walking Distance after Supervised Exercise Training Is Related to Changes in Quality of Life in Patients with Lower Extremity Peripheral Artery Disease. J. Clin. Med..

[34] ATS Committee on Proficiency Standards for Clinical Pulmonary Function Laboratories (2002). ATS statement: Guidelines for the six-minute walk test. Am. J. Respir. Crit. Care. Med..

[35] Borg G.A. (1982). Psychophysical bases of perceived exertion. Med. Sci. Sports Exerc..

[36] Calanca L., Lanzi S., Ney B., Berchtold A., Mazzolai L. (2020). Multimodal Supervised Exercise Significantly Improves Walking Performances Without Changing Hemodynamic Parameters in Patients with Symptomatic Lower Extremity Peripheral Artery Disease. Vasc. Endovasc. Surg..

[37] Ney B., Lanzi S., Calanca L., Mazzolai L. (2021). Multimodal Supervised Exercise Training Is Effective in Improving Long Term Walking Performance in Patients with Symptomatic Lower Extremity Peripheral Artery Disease. J. Clin. Med..

[38] Dadashi F., Mariani B., Rochat S., Büla C.J., Santos-Eggimann B., Aminian K. (2013). Gait and Foot Clearance Parameters Obtained Using Shoe-Worn Inertial Sensors in a Large-Population Sample of Older Adults. Sensors.

[39] Mariani B., Hoskovec C., Rochat S., Büla C., Penders J., Aminian K. (2010). 3D gait assessment in young and elderly subjects using foot-worn inertial sensors. J. Biomech..

[40] Wüest S., Massé F., Aminian K., Gonzenbach R., De Bruin E.D. (2016). Reliability and validity of the inertial sensor-based Timed “Up and Go” test in individuals affected by stroke. J. Rehabil. Res. Dev..

[41] Bregou Bourgeois A., Mariani B., Aminian K., Zambelli P.Y., Newman C.J. (2014). Spatiotemporal gait analysis in children with cerebral palsy using, foot-worn inertial sensors. Gait Posture.

[42] Mariani B., Jimenez M.C., Vingerhoets F.J., Aminian K. (2013). On-shoe wearable sensors for gait and turning assessment of patients with Parkinson’s disease. IEEE Trans. Biomed. Eng..

[43] McDermott M.M., Tian L., Criqui M.H., Ferrucci L., Conte M.S., Zhao L., Li L., Sufit R., Polonsky T.S., Kibbe M.R. (2021). Meaningful change in 6-minute walk in people with peripheral artery disease. J. Vasc. Surg..

[44] Gardner A.W., Montgomery P.S., Wang M. (2018). Minimal clinically important differences in treadmill, 6-minute walk, and patient-based outcomes following supervised and home-based exercise in peripheral artery disease. Vasc. Med..

[45] Beekman E., Mesters I., Hendriks E.J., Klaassen M.P., Gosselink R., Van Schayck O.C., De Bie R.A. (2013). Course length of 30 metres versus 10 metres has a significant influence on six-minute walk distance in patients with COPD: An experimental crossover study. J. Physiother..

[46] Sandberg A., Cider Å., Jivegård L., Nordanstig J., Wittboldt S., Bäck M. (2020). Test-retest reliability, agreement, and minimal detectable change in the 6-minute walk test in patients with intermittent claudication. J. Vasc. Surg..

[47] Pipinos I.I., Judge A.R., Selsby J.T., Zhu Z., Swanson S.A., Nella A.A. (2007). The myopathy of peripheral arterial occlusive disease: Part 1. Functional and histomorphological changes and evidence for mitochondrial dysfunction. Vasc. Endovasc. Surg..

[48] Pipinos I.I., Judge A.R., Selsby J.T., Zhu Z., Swanson S.A., Nella A.A. (2008). The myopathy of peripheral arterial occlusive disease: Part 2. Oxidative stress, neuropathy, and shift in muscle fiber type. Vasc. Endovasc. Surg..

[49] Dziubek W., Stefańska M., Bulińska K., Barska K., Paszkowski R., Kropielnicka K., Jasiński R., Rachwalik A., Woźniewski M., Szuba A. (2020). Effects of Physical Rehabilitation on Spatiotemporal Gait Parameters and Ground Reaction Forces of Patients with Intermittent Claudication. J. Clin. Med..

[50] Nascimento L.R., de Oliveira C.Q., Ada L., Michaelsen S.M., Teixeira-Salmela L.F. (2015). Walking training with cueing of cadence improves walking speed and stride length after stroke more than walking training alone: A systematic review. J. Physiother..

