# Supplementary material for: Supervised Exercise Training Improves 6 min Walking Distance and Modifies Gait Pattern during Pain-Free Walking Condition in Patients with Symptomatic Lower Extremity Peripheral Artery Disease
Source: Sensors (Basel). 2021 Nov 30;21(23):7989. doi: 10.3390/s21237989 (PMC8659842; doi:10.3390/s21237989)
Supplement: Supplementary file 1 [file sensors-21-07989-s001.zip › sensors-1413018-supplementary.pdf]

Supplementary materials

The details of the estimation of the spatiotemporal gait and foot kinematics parameters were previously presented by Dadashi et al. (1). We reported here below these calculations quoting their Methods (1).

First, the sensors were turned on with the patients in the standing position and were synchronized wirelessly. The recorded data were sampled at a frequency of 200 Hz and then low-pass filtered at 17 Hz.

(1) Estimation of the stance temporal parameters

The stance period at cycle k was calculated as follow:

$$\Delta T_{St}^{k} = t(TO^{k}) - t(HS^{k})$$

where: $t(.)$ is the instant when the temporal event occurred; TO is the Toe-Off; HS is the Heel-Strike.

The duration of the three inner-stance phases, the loading response (Load), the foot-flat (ff), and the push-off (Push) phases have been determined as follow:

$$\Delta T_{\text{Load}}^{k} = t(TS^{k}) - t(HS^{k})$$
$$\Delta T_{ff}^{k} = t(HO^{k}) - t(TS^{k})$$
$$\Delta T_{\text{Push}}^{k} = t(TO^{k}) - t(HO^{k})$$

where: TS is the Toe-Strike; HO is the Heel-Off.

Considering N_S as the number of steps at each measurement, the gait cycle duration $\left(\Delta T_{Cyc}^{k}\right)$ and the stride frequency were calculated:

$$\Delta T_{Cyc}^{k} = t(HS^{k}) - t(HS^{k-1})$$
$$\text{Stride frequency } = 60N_s / \sum_{k=1}^{N_s} \Delta T_{Cyc}^{k}$$

(2) Estimation of the spatial gait parameters

At instant $i = 1,2,\ldots,N$ of cycle k, the orientation of the foot relative to the global frame $R^{k}(i)$ was calculated by strap-down integration of the angular velocity vector (2). The initial orientation $R^{k}(0)$ was obtained by using the acceleration signal during motionless period of ΔT_{ff}^{k}. Subsequently, the gravity-free acceleration in the global frame ($\vec{a}_{GF}$) was calculated:

$$\vec{a}_{GF}^{k}(i) = \vec{a}^{k}(i) \cdot R^{k}(i) - \vec{g}, \vec{g} = [0,0,1]$$

Then, foot velocity ($\vec{v}_{GF}^{k}$) and position ($\vec{p}_{GF}^{k}$) were estimated from the numerical integration of $\vec{a}_{GF}$. At each cycle, spatial gait descriptors of the foot trajectory were extracted. The stride velocity is defined as the average of the foot velocity in the horizontal plane of walking during two successive foot-flats. The stride length was defined as the linear distance between two successive foot-flat positions in the frontal axis. The swing width was defined as the maximum lateral deviation of the foot trajectory during the swing phase. Finally, the path length is defined as the length of 3D curve $\vec{p}_{GF}^{k}$ normalized by the stride length.

(3) Estimation of the foot kinematic parameters

A 2D kinematic model has been used to estimate the inertial measurement unit (IMU) position relative to the foot in order to calculate the heel and toe trajectories. By defining the IMU distance with regard to the heel and toe as three unknowns *a, b, and c,* the vertical trajectory of the IMU (Z_{IMU}^{k}), heel $\left(Z_{Heel}^{k}\right)$ and toe (Z_{Toe}^{k}) were extracted as follow:

$$Z_{IMU}^{k}(i) = Z_{GF}^{k}(i) + b$$
$$Z_{Toe}^{k}(i) = Z_{GF}^{k}(i) - b \cdot \cos\big(\theta_Y(t)\big) + c \cdot \sin\big(\theta_Y(t)\big)$$
$$Z_{Heel}^{k}(i) = Z_{GF}^{k}(i) - b \cdot \cos\big(\theta_Y(t)\big) - a \cdot \sin\big(\theta_Y(t)\big)$$

where: $Z_{GF}^{k}(i)$ is the vertical component of the foot position ($\vec{p}_{GF}^{k}$); θ_Y is the pitch angle at the heel strike. Constraints in the following equation were adopted to estimate a, b and c and toe and heel trajectories:

$$\begin{cases} Z_{\text{Toe}}^{k}\,(TO^{k}) = 0 \\ Z_{\text{Heel}}^{k}\,(HS^{k}) = 0 \\ a + c = \text{ Shoe Size} \end{cases}$$

Considering all cycles, a least square solution was used to estimate a, b *and* c and the following ground contact constraints were considered in order to correct the 2D trajectory of the toe and heel.

$$\begin{cases} \forall t \in \left[T_{ff}^{k}, TO^{k}\right]: Z_{Toe}^{k}(t) = 0 \\ \forall t \in \left[HS^{k}, T_{ff}^{k}\right]: Z_{Heel}^{k}(t) = 0 \end{cases}$$

The corrections in the above equation do not lead to discontinuities in the toe and heel trajectories since the equations have been solved with null toe and heel elevation at TO^{k} and HS^{k} instants.

References

1. Dadashi F, Mariani B, Rochat S, Bula CJ, Santos-Eggimann B, Aminian K. Gait and foot clearance parameters obtained using shoe-worn inertial sensors in a large-population sample of older adults. Sensors (Basel) 2014;14:443-57.
2. Mariani B, Hoskovec C, Rochat S, Bula C, Penders J, Aminian K. 3D gait assessment in young and elderly subjects using foot-worn inertial sensors. J Biomech 2010;43:2999-3006.
